# Increased food availability raises eviction rate in a cooperative breeding mammal

**DOI:** 10.1098/rsbl.2016.0961

**Published:** 2017-04-12

**Authors:** C. Dubuc, S. English, N. Thavarajah, B. Dantzer, S. P. Sharp, H. C. Spence-Jones, D. Gaynor, T. H. Clutton-Brock

**Affiliations:** 1 Department of Zoology, University of Cambridge, Cambridge, UK; 2 Kalahari Meerkat Project, Kuruman River Reserve, Kuruman, South Africa; 3 Mammal Research Institute, Department of Zoology and Entomology, University of Pretoria, Pretoria, South Africa

**Keywords:** dispersal, food competition, breeding competition, meerkats

## Abstract

In group-living mammals, the eviction of subordinate females from breeding groups by dominants may serve to reduce feeding competition or to reduce breeding competition. Here, we combined both correlational and experimental approaches to investigate whether increases in food intake by dominant females reduces their tendency to evict subordinate females in wild meerkats (*Suricata suricatta*). We used 20 years of long-term data to examine the association between foraging success and eviction rate, and provisioned dominant females during the second half of their pregnancy, when they most commonly evict subordinates. We show that rather than reducing the tendency for dominants to evict subordinates, foraging success of dominant females is positively associated with the probability that pregnant dominant females will evict subordinate females and that experimental feeding increased their rates of eviction. Our results suggest that it is unlikely that the eviction of subordinate females serves to reduce feeding competition and that its principal function may be to reduce reproductive competition. The increase in eviction rates following experimental feeding also suggests that rather than feeding competition, energetic constraints may normally constrain eviction rates.

## 1. Introduction

In group-living mammals, adult females may leave their natal groups voluntarily when food competition increases (e.g. African lions, *Panthera leo*, California ground squirrels, *Otospermophilus beecheyi* [1]), while in some cooperative breeders, dispersal is commonly imposed by breeding females who commonly evict subordinate females from the group (e.g. meerkats, *Suricata suricatta*, banded mongooses, *Mungos mungo* [1,2]). The eviction of subordinates may benefit dominants either by reducing feeding competition or by reducing the risk that they will attempt to breed or to challenge dominants for the breeding role [1–3]. As yet, few attempts have been made to distinguish between these possibilities. Here, we use a combination of long-term records of the behaviour of individuals and experiment in which we increased the food intake of dominant females in wild meerkats (*S. suricatta*) to investigate whether foraging success affects the tendency of dominants to evict subordinates. We also investigated whether foraging success affects the timing of eviction during pregnancy.

Meerkats live in groups of 2–50 where reproduction is monopolized by a dominant pair that breed up to three or four times year, though subordinate females breed occasionally [1,4]. Pregnant dominant females evict subordinate females from the group when they reach an age when their weight approaches that of dominant females and the frequency with which they attempt to breed increases [3]. Evictions are frequently occurring in large groups and involving older and heavier subordinate females, which are the ones most likely to breed [3,5]. Subordinate females that have been evicted from their group by the dominant female often attempt to return, both before and after the dominant gives birth [3]. Those that try to return before dominants give birth are usually evicted again; those that try afterwards may be allowed to rejoin the group, though they are then usually evicted again during the next breeding event [3]. The timing of evictions suggests that evicting older subordinate females may serve to reduce the risk that they will kill the dominant female's pups. Subordinate breeding has substantial costs to the success of dominants: pregnant subordinates commonly kill offspring born to dominant females shortly after birth [6] and, if litters born to dominants and subordinates are reared at the same time, the growth of pups born to dominants is reduced [7]. However, the presence of positive correlations between group size and the probability of eviction [3] suggests that eviction may also serve to reduce feeding competition.

If evicting subordinate females serves to reduce feeding competition and increase access to resources for dominant females, improvements in their foraging success should lead to increased tolerance towards subordinates and reduced rates of eviction. By contrast, if eviction serves to reduce breeding competition and the risk of infanticide, no consistent relationship between the dominants female's foraging success and the eviction of subordinate females would be expected—unless the probability that dominants will evict subordinates is constrained by their access to resources, when a positive relationship between foraging success and rates of eviction would be expected.

## 2. Material and methods

All data used in our analyses were collected at the Kuruman River Reserve, South Africa, as part of the long-term Kalahari Meerkat Project (KMP) which has followed more than 60 different groups of wild meerkats over 20 years [4]. Details of the measurement of life-history events (pregnancy, birth, eviction) and weights are provided in the electronic supplementary material. All animals in our study groups were individually recognizable and habituated to close observation by humans. They were also trained to step onto an electronic balance in return for small rewards of hard-boiled egg to collect individual weight three times a day (at dawn, around midday and at dusk) when groups were visited. The foraging success of pregnant dominant females was calculated as their average weight gained during the first 3 h of foraging in the morning [8]. Since subordinate females never leave groups voluntarily [1,9], we considered as eviction all instances where subordinate females over nine months old (minimal age at reproduction [9]) suddenly disappeared from their groups whilst the dominant female was pregnant. Multiple evictions of the same subordinate females were considered as separate events, though we also measured the number of subordinate females evicted. Because dominant females’ propensity to evict subordinate females might be constrained by the number of helpers available to contribute to alloparental care [10], we also counted the number of subordinate males, using the same age cut-off (see electronic supplementary material).

We initially investigated whether variation in the probability that pregnant dominant females would evict subordinates was correlated with their own foraging success. Since subordinate females are seldom evicted unless the dominant female is pregnant and older subordinate females have usually been permanently evicted by the mid-point of each breeding seasons, we extracted records of the frequency of eviction for all pregnancies that took place in the study population during the first half of the breeding season between 1997 and 2015. Cases where dominants miscarried and pregnancies that took place in groups without subordinate females were excluded. In total, we extracted data for 154 pregnancies of 64 dominant females who lived in 36 different groups of the population over 18 years, with 3.82 ± 2.27 (mean ± s.d.) pregnancies per female.

We also experimentally provisioned 10 dominant females in 10 different groups during the second half of their pregnancy, when evictions take place, with one hen's egg per day (one half in the morning, one half in the evening; see the electronic supplementary material). All trials took place in the first part of the rainy season and include pregnancies that ended in August–November of two consecutive years (2011 and 2012), with five trials being conducted in each year. As controls, we selected all other successful dominant pregnancies that ended in August–November 2011 and 2012 (*N* = 8 pregnancies from six different females), as well as pregnancies involving females used in the experiment that ended in August–November the year before or after the year when they were experimentally fed (*N* = 10 pregnancies of seven dominant females; see details in the electronic supplementary material). This gave a total of 28 pregnancies for 16 females of 14 groups, with 1.75 ± 0.19 pregnancies per female (2.00 ± 0.26 for fed subjects).

We used linear mixed models (LMMs) to examine whether dominant females’ foraging success or experimental feeding (fixed effects) influenced the number of evictions, the number of subordinate females evicted and the timing of eviction (response variables). In most models, we set the ‘number of subordinate females’ and ‘number of subordinate males’ as fixed terms, which were combined into ‘number of subordinates’ in the model setting ‘timing of eviction’ as response variable (see the electronic supplementary material). In all models, ‘female identity’, ‘group identity’, ‘year’ and ‘month’ (nested in year) were set as random factors. In the correlational analyses, to meet the assumptions of the model, we log-transformed ‘number of evictions’ and square-root-transformed ‘number of subordinate females evicted’, log-transformed ‘foraging success’ in models setting ‘number of evictions’ and ‘number of subordinate females evicted’, and log-transformed all the other fixed effects. In the experimental analyses, we also included ‘treatment’ (fed versus controls) as a fixed effect in addition to the fixed and random effects described above, and also included ‘rainfall’ to account for the potential effect of variation in natural food availability on dominant females’ access to food (see the electronic supplementary material). ‘Rainfall’ was log-transformed, but no other transformation was required. Finally, to examine whether experimental feeding improved dominant females’ body condition, we set ‘weight gain’ over the course of pregnancy (see electronic supplementary material) as the response variable, ‘treatment’ and log-transformed ‘rainfall’ as fixed effects, and used the same random effects as above. Since ‘number of evictions’, ‘number of females evicted’ and ‘rainfall’ could be nil, we added the value ‘1’ to all entries to allow transformation. All statistical analyses were computed with IBM SPSS Statistics 23. *α* levels were set at 0.05 and analyses were two-tailed.

## 3. Results

**Figure 1.**
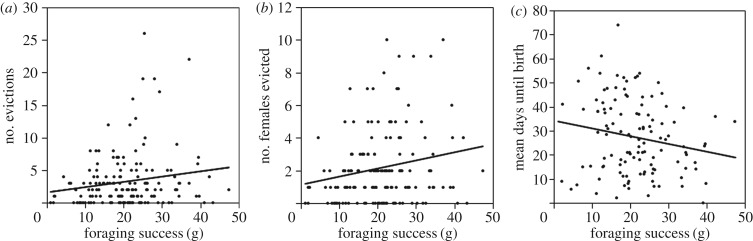
Association between average daily foraging success of pregnant dominant females and the total number of evictions (*a*), number of females evicted (*b*) and timing of eviction (*c*).

The probability that dominant females would evict subordinates was significantly positively correlated with their average foraging success: dominant females who gained more weight while foraging conducted more eviction events and evicted more females from their group (figure 1(*a*,*b*) and table 1). Foraging success also affected the timing of eviction: well-fed females evicted subordinate females on average closer to their own parturition (figure 1*c*).

**Table 1.** Results from LMMs testing for the effect of foraging success on number of evictions, number of females evicted and timing of eviction by dominant females.

	number of evictions				number of females evicted					timing of eviction			
	estimate ± s.e.	d.f. (numerator, denominator)	*F*-value	*p*-value	estimate ± s.e.	d.f. (numerator, denominator)	*F*-value	*p*-value		estimate ± s.e.	d.f. (numerator, denominator)	*F*-value	*p*-value
*fixed effects*									*fixed effects*				
intercept	−0.50 ± 0.16	1, 145.396	9.985	0.002	0.37 ± 0.21	1, 147.283	0.314	0.576	intercept	39.67 ± 7.81	1, 114.916	25.797	<0.001
foraging success	0.21 ± 0.10	1, 139.326	4.576	0.034	0.42 ± 0.16	1, 146.319	7.269	0.008	rainfall	−0.37 ± 0.17	1, 89.225	4.648	0.034
no. subordinate females	0.78 ± 0.10	1, 132.161	67.452	<0.001	1.38 ± 0.15	1, 140.962	82.991	<0.001	no. subordinates	−4.26 ± 6.59	1, 108.763	0.418	0.519
no. subordinate males	0.22 ± 0.13	1, 137.021	3.170	0.077	0.43 ± 0.19	1, 143.598	4.976	0.027					
*random factors*									*random factors*				
ID	0.01 ± 0.01	—	—	—	0.00 ± 0.00	—	—	—	ID	0.00 ± 0.00	—	—	—
group	0.00 ± 0.01	—	—	—	0.00 ± 0.00	—	—	—	group	1.31 ± 13.70	—	—	—
year	0.01 ± 0.01	—	—	—	0.01 ± 0.02	—	—	—	year	0.00 ± 0.00	—	—	—
month	0.01 ± 0.01	—	—	—	0.03 ± 0.02	—	—	—	month	38.94 ± 30.47	—	—	—

**Figure 2.**
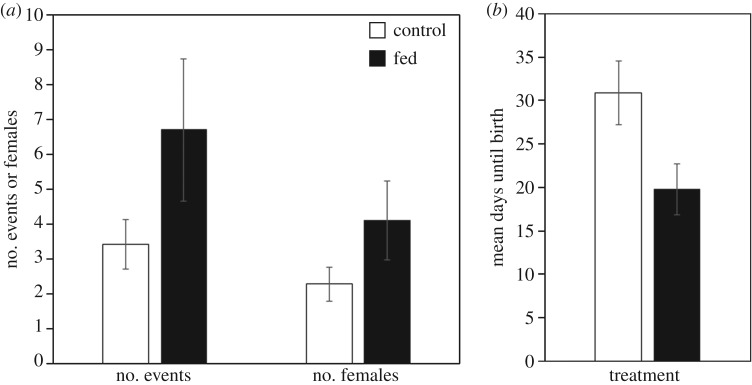
Effect of experimental supplementation of the diet of pregnant dominant females (black) on the total number of eviction events and number of females evicted (*a*) and on the timing of eviction (*b*) compared with controls (white). Values represent mean ± s.e.m.

Our experiment provided additional evidence of this positive relationship: dominant females that were experimentally fed evicted more subordinates, in more separate eviction events, and did so closer to parturition than control females (figure 2 and table 2), although they did not gain more weight (*F*_1,25.922_ = 1.309, *p* = 0.263).

**Table 2.** Results from LMMs comparing the number of evictions, number of females evicted and timing of eviction between fed and control pregnant dominant females.

	number of evictions				number of females evicted					timing of eviction			
	estimate ± s.e.	d.f. (numerator, denominator)	*F*-value	*p*-value	estimate ± s.e.	d.f. (numerator, denominator)	*F*-value	*p*-value		estimate ± s.e.	d.f. (numerator, denominator)	*F*-value	*p*-value
*fixed effects*									*fixed effects*				
intercept	2.07 ± 0.23	1, 17.615	0.004	0.948	0.90 ± 1.13	1, 26.000	0.037	0.849	intercept	25.96 ± 8.55	1, 18.000	15.163	0.001
treatment	−3.86 ± 1.63	1, 25.169	5.585	0.026	−2.22 ± 0.88	1, 26.000	6.376	0.018	treatment	14.24 ± 6.35	1, 18.000	5.035	0.038
rainfall	−1.83 ± 2.44	1, 25.310	0.563	0.460	−0.63 ± 1.31	1, 26.000	0.229	0.636	rainfall	−8.02 ± 10.93	1, 18.000	0.538	0.473
no. subordinate females	0.79 ± 0.36	1, 25.272	4.807	0.038	0.58 ± 0.19	1, 26.000	9.142	0.006	no. subordinates	−0.18 ± 0.47	1, 18.000	0.158	0.696
no. subordinate males	0.24 ± 1.54	1, 25.093	2.598	0.120	0.14 ± 0.09	1, 26.000	2.563	0.121					
*random factors*									*random factors*				
ID	0.00 ± 0.00	—	—	—	0.00 ± 0.00	—	—	—	ID	0.00 ± 0.00	—	—	—
group	0.00 ± 0.00	—	—	—	0.00 ± 0.00	—	—	—	group	0.00 ± 0.00	—	—	—
year	2.05 ± 3.34	—	—	—	0.00 ± 0.00	—	—	—	year	0.00 ± 0.00	—	—	—
month	0.00 ± 0.00	—	—	—	0.00 ± 0.00	—	—	—	month	0.00 ± 0.00	—	—	—

## 4. Discussion

Our aim was to investigate whether food competition stimulates the eviction of subordinate females by dominants in wild Kalahari meerkats. Combining correlational and experimental approaches, we show that increased foraging success does not reduce the tendency of dominant females to evict subordinate females: on the contrary, well-fed dominant females were more likely to evict subordinate females, indicating that there is a causal relationship between the foraging success of dominant females and their tendency to evict subordinate females. Our results also show that increased food intake led to evictions taking place closer to parturition, supporting the view that the proximate function of eviction is to avoid breeding competition in meerkats.

Our results raise the question of why increased food intake should increase the probability of evictions. One possible explanation is that dominant females’ readiness to evict subordinates is constrained by the energetic costs or the physical risks associated with the process of eviction [7]. Possible energetic costs of eviction include those associated with increased androgen and glucocorticoid levels [11,12] generated by competitive contexts, as well as decreased investment of time in foraging and antipredator activity [13]. Low food availability might constrain the opportunity for dominant females to evict subordinate females by raising the time necessary for foraging or increasing the average physical distance between dominant females and likely evictees during foraging bouts. The absence of any weight gain in experimentally fed dominant females is consistent with the suggestion that the process of eviction has energetic costs, suggesting that the extra energy acquired may have been invested towards eviction rather than condition.

Comparison between our results and recent studies of banded mongooses suggests that the effects of variation in food availability on dispersal may differ across breeding systems. In banded mongooses—where multiple members of both sexes breed regularly—low food availability (estimated using rainfall as a proxy) appears to increase the risk of eviction of subordinates by breeders in this species [14], though the role of foraging success has not been measured directly. Increased rates of dispersal when food availability is low have also been documented in several social mammals where young females disperse voluntarily [1], suggesting that the positive relationship between the condition of dominant females and the incidence of eviction in meerkats may reflect the large power asymmetries between females typical of singular cooperative breeders.

## Supplementary Material

Method details

## Supplementary Material

Dataset

## Data accessibility

Data are available as the electronic supplementary material.
