# Supplementary material for: Increased food availability raises eviction rate in a cooperative breeding mammal
Source: Biol Lett. 2017 Apr 12;13(4):20160961. doi: 10.1098/rsbl.2016.0961 (PMC5414692; doi:10.1098/rsbl.2016.0961)
Supplement: Method details [file rsbl20160961supp1.docx]

**Food availability increases eviction in a cooperative breeding mammal**

Dubuc, C., English, S., Thavarajah, N., Dantzer, B., Sharp, S.P., Spence-Jones, H.C., Gaynor, D. & Clutton-Brock, T.H.

**SUPPLEMENTAL MATERIAL 1**

*Study population*

Meerkats (*Suricata suricatta*) are cooperative breeding mongooses that live in groups of 2-50 females in which one breeding pair monopolizes reproduction, although subordinate females occasionally breed during the rainy season (Clutton-Brock 2016). Data used here were collected on wild meerkats of the Kalahari Meerkat Project (KMP) at the Kuruman River Reserve, South Africa, between 1997 and 2015. Typically, 10-15 groups were followed in any year, but in total, more than 60 different groups were studied in the 18-year-long period. Groups were visited 2–4 times per week, when data on basic life history parameters (pregnancy, birth, eviction, survival) and body mass were collected.

*Life history data*

Pregnancies last around 70 days in meerkats (Sharp et al. 2013). Pregnancies could be detected at mid-point based on the sudden rise in body weight and swelling of the abdomen, while the end of pregnancy was assessed from on a sudden loss in weight and abdomen distention (Sharp et al. 2013). Since mating is seldom observed, conception dates of live-birth were inferred by counting back 70 days from parturition. Since new-born pups are kept in an underground burrow, live birth was confirmed if a group member stayed behind as a babysitter in the morning instead of going foraging as or if the dominant female showed visual signs of suckling (wet or tapped hair around the nipples).

We considered as *evictions* all instances where subordinate females suddenly disappeared from the group, whether they were observed or not, because subordinate females never leave groups willingly (Clutton-Brock et al. 1998, 2001). In 99.3% the cases, subordinate females that suddenly disappeared from their groups during the pregnancy of dominant females were seen subsequently (either outside their group or upon their return in their natal group), indicating that almost all disappearances of subordinate females during the pregnancy of dominants are the result of eviction rather than of predation or other sources of mortality. Multiple evictions of the same subordinate females were considered as separated events when calculating the *number of eviction events*, but not when calculating the *number of subordinate females evicted.* We also considered cases when subordinate females successfully reentered groups and disappeared as independent evictions, unless they took place the same day. As a result, only one eviction per subordinate female per day was considered in the analyses. When computing the *number of subordinate females*, we considered all females who had reached 9 months, the minimal age at reproduction (Bell et al. 2014). This was considered as a confounding factor affecting the number of females available to be evicted. Since the propensity to evict is likely to be affected by the number of helpers available (see Clutton-Brock et al. 2010, Cant et al. 2010, Bell et al. 2014), we also counted the *number of subordinate males* present in the group; we used the same age cut off as for females as both sexes reach sexual maturity at the same age (see Mares et al. 2012). The number of subordinate females and number of subordinate males were averaged across the entire pregnancy. The two variables were not significantly correlated to each other (Spearman rho=0.316, p=0.084, N=31), reducing any problem of collinearity in the statistical analyses.

*Estimation of foraging success*

Habituated individuals were trained to step onto a balance in return for a small reward of hard-boiled egg crumbs, allowing us to record body mass (± 1 g) 2-4 days per week throughout their lives. Individuals were weighed three times over the course of the day: upon emergence from the burrow in the morning before foraging starts (morning weight), after three hours of foraging (lunch weight) and at the end of the day upon returning to a burrow for the night (evening weight). The *foraging success* of dominant females was assessed using morning-to-lunch weight gain by subtracting morning weight from lunch weight over the 3-hour morning foraging period for each day of observation, and averaged across pregnancy.

In some analyses, we used *total rainfall* as a proxy of natural food availability. This was measured by adding up amount in millilitres measured at the field site weather station for the entire duration of the pregnancy. Rainfall is positively correlated with food intake and condition in meerkats of the study population (Gaynor et al in prep). Morning-to-lunch weight gain and total rainfall were positively correlated across 132 non-experimental pregnancies for which information on both variable was available (Spearman’s r_s_=0.370, p<0.001, N=132).

*Feeding experiment*

Experimental feeding took place in the first months of the breeding seasons 2011 and 2012, and included live pregnancies that ended between August and November of the same years, inclusively. Dominant females were fed following an established procedure (e.g. Russell et al. 2004, Huchard et al. 2016). We fed dominant females in the second half of their pregnancy, when evictions can take place. Since evictions can start in the 6^th^ week of 10-week (70 days) long pregnancy (Clutton-Brock et al. 1998) and meerkat females can become pregnant immediately after parturition (Russell et al. 2003), the experimental feeding commenced six weeks following the previous birth or miscarriage (see definition below), and continued until the birth of the present litter. Subject dominant females received on average 18.3 ± 11.4 eggs over 34.3 ± 21.3 days.

As control pregnancies, we selected all dominant females’ live pregnancies in groups containing at least one sexually mature subordinate female that ended in August-November that fitted in either of these two categories: (i) pregnancies of non-fed dominant females in other groups of the population in the time period where the experiment took place (2011-2012) (N=8 pregnancies from 6 different females) and (ii) pregnancies of the fed subjects of the experiment that took place in the same time window but the year before or after they were fed (10 pregnancies from 7 different females). Control pregnancies were not available for three of the fed dominant females: in one case, the female was only dominant for one breeding year and was dead in the following year time-period; in the two remaining ones, the dominant female either did not breed successfully between August-November of the previous or subsequent year, or there were no sexually-mature subordinate females in the group at that time. The final dataset consisted in a total of 28 pregnancies for 16 females of 14 groups, with 1.75 ± 0.19 cycles pregnancies per female (2.00 ± 0.26 for fed subjects).

To examine whether experimental feeding improved dominant females’ body condition, we compared the weight gained over the course of pregnancy between fed pregnancies and controls. Weight gain was calculated as the difference between the average morning weight over the week before conception and average morning weight over the last week of pregnancy.

*References*

Bell MBV, Cant MA, Borgeaud C, Thavarajah N, Samson J, Clutton-Brock TH 2014 Suppressing subordinate reproduction provides benefits to dominants in cooperative societies of meerkats. *Nature Communications*, 5:4499.

Cant MA, Hodge SJ, Bell MBV, Gilchrist JS, Nichols HJ. 2010. Reproductive control via eviction (but not the threat of eviction) in banded mongooses. *Proc R Soc Lond B Biol Sci* 277, 2219–2226.

Clutton-Brock TH. 2016 *Mammal Societies*. John Wiley and Sons.

Clutton-Brock TH, Brotherton PNM, Smith R, McIlrath GM, Kansky R, Daynor D, O’Riain, Skinner, JD 1998 Infanticide and expulsion of females in cooperative mammal. *Proc Roy Soc Lond B* 265, 2291-2295.

Clutton-Brock TH, Brotherton PNM, Russell AF, O’Riain MJ, Gaynor D, Kansky R, Griffin A, Manser M, Sharpe L, McIlrath GM, Small TM, Moss A, Monfort S 2001 Cooperation, control, and concession in meerkat groups. *Science* 291, 478-481.

Clutton-Brock TH, Hodge SJ, Flower TP, Spong GF, Young AJ. 2010. Adaptive suppression of subordinate reproduction in cooperative mammals. *Am Nat* 176, 664–673.

Huchard E, English S, Bell MBV, Thavarajah N, Clutton-Brock TH 2016 Competitive growth in a cooperatively breeding mammal. *Nature* 533, 532-534.

Mares R, Young AJ, Clutton-Brock TH. 2012 Individual contributions to territory defense in a cooperative breeder: weighing up the benefits and costs. *Proc R Soc Lond B* 279, 3989-3995.

Russell, A.F., Brotherton, P.N.M., McIlrath, G.M., Sharpe, L.L. and Clutton-Brock, T.H. (2003) Breeding success in cooperative meerkats: effects of helper number and maternal state. Behavioural Ecology 14: 486-492.

Russell AF, Young AJ, Spong G, Jordan NR, Clutton-Brock TH. 2007. Helpers increase the reproductive potential of offspring in cooperative meerkats. Proc Roy Soc Lond B 274, 513-520.

Sharp SP, English S, Clutton-Brock TH. 2013. Maternal investment during pregnancy in wild meerkats. *Evol Eco*l 27, 1033.
