# Supplementary material for: Increased food availability raises eviction rate in a cooperative breeding mammal
Source: Biol Lett. 2017 Apr 12;13(4):20160961. doi: 10.1098/rsbl.2016.0961 (PMC5414692; doi:10.1098/rsbl.2016.0961)
Supplement: Dataset [file rsbl20160961supp2.docx]

**Food availability increases eviction in a cooperative breeding mammal**

Dubuc, C., English, S., Thavarajah, N., Dantzer, B., Sharp, S.P., Spence-Jones, H.C., Gaynor, D. & Clutton-Brock, T.H.

Table S1. Dataset used for the correlational analyses using LMMs to test the effect of foraging success on a series of proxies of probability of eviction its timing and the characteristics of targeted subordinate females.

| Dominant female ID | Group | Month | Year | Foraging success (g) | Number of Subordinate females | Number of Subordinate males | Number of helpers | Number of evictions | Number of females evicted | Day until birth |
| --- | --- | --- | --- | --- | --- | --- | --- | --- | --- | --- |
| 529 | E | 9 | 1997 | 17.0 | 2.0 | 2.9 | 4.9 | 0 | 0 |  |
| 258 | JJ | 10 | 1997 | 24.8 | 3.3 | 2.1 | 5.5 | 1 | 1 | 3.0 |
| 259 | Y | 11 | 1997 | 19.3 | 0.9 | 6.9 | 7.7 | 0 | 0 |  |
| 1314 | V | 11 | 1997 | 29.9 | 2.0 | 4.0 | 6.1 | 2 | 1 | 28.0 |
| 256 | L | 11 | 1997 | 27.9 | 5.2 | 6.6 | 11.8 | 1 | 1 | 27.0 |
| 530 | E | 9 | 1998 | 8.6 | 1.9 | 6.7 | 8.6 | 0 | 0 |  |
| 1314 | V | 9 | 1998 | 26.0 | 7.7 | 7.4 | 15.1 | 5 | 4 | 14.0 |
| 395 | D | 10 | 1998 | 17.5 | 2.4 | 8.9 | 11.3 | 3 | 3 | 18.0 |
| 256 | L | 10 | 1998 | 34.4 | 6.9 | 6.6 | 13.5 | 1 | 1 | 21.0 |
| 259 | Y | 10 | 1998 | 20.9 | 3.2 | 8.5 | 11.6 | 2 | 2 | 19.0 |
| 894 | X | 11 | 1998 | 21.7 | 1.0 | 2.9 | 3.9 | 0 | 0 |  |
| 40 | W | 11 | 1998 | 23.2 | 3.9 | 5.0 | 8.9 | 2 | 2 | 44.0 |
| 40 | W | 10 | 1999 | 7.6 | 5.5 | 5.5 | 11.0 | 2 | 2 | 15.0 |
| 37 | F | 10 | 1999 | 22.5 | 4.0 | 6.3 | 10.3 | 1 | 1 | 12.0 |
| 530 | E | 10 | 1999 | 12.8 | 3.9 | 4.2 | 8.1 | 1 | 1 | 15.0 |
| 1314 | V | 10 | 1999 | 23.7 | 0.9 | 8.3 | 9.1 | 1 | 1 | 13.0 |
| 1320 | NN | 10 | 1999 | 12.4 | 4.9 | 3.9 | 8.8 | 0 | 0 |  |
| 256 | L | 11 | 1999 | 23.5 | 4.5 | 4.4 | 8.9 | 5 | 5 | 24.8 |
| 894 | X | 11 | 1999 | 22.4 | 1.5 | 2.5 | 4.0 | 1 | 1 | 31.0 |
| 395 | D | 11 | 1999 | 20.1 | 0.8 | 10.3 | 11.1 | 0 | 0 |  |
| 259 | Y | 11 | 1999 | 24.6 | 6.7 | 7.0 | 13.7 | 6 | 5 | 36.7 |
| 45 | UU | 11 | 1999 | 10.0 | 2.6 | 3.4 | 6.0 | 0 | 0 |  |
| 37 | F | 9 | 2000 | 4.5 | 2.8 | 5.2 | 8.1 | 4 | 4 | 5.8 |
| 395 | D | 9 | 2000 | 15.0 | 1.5 | 7.2 | 8.7 | 3 | 2 | 19.7 |
| 40 | W | 9 | 2000 | 13.2 | 5.9 | 6.0 | 11.8 | 4 | 3 | 18.8 |
| 44 | UU | 9 | 2000 | 14.8 | 2.2 | 3.0 | 5.3 | 3 | 2 | 39.3 |
| 259 | Y | 10 | 2000 | 23.4 | 7.4 | 10.7 | 18.1 | 6 | 4 | 31.0 |
| 256 | L | 10 | 2000 | 11.4 | 3.2 | 9.0 | 12.2 | 3 | 2 | 29.3 |
| 1319 | RR | 11 | 2000 | 23.6 | 1.8 | 6.1 | 7.9 | 1 | 1 | 12.0 |
| 1324 | V | 11 | 2000 | 28.2 | 2.2 | 14.0 | 16.2 | 1 | 1 | 33.0 |
| 37 | F | 11 | 2000 | 34.5 | 3.8 | 5.8 | 9.6 | 3 | 3 | 14.3 |
| 259 | Y | 9 | 2001 | 12.8 | 5.3 | 13.9 | 19.2 | 8 | 7 | 41.8 |
| 915 | L | 9 | 2001 | 13.7 | 8.5 | 7.0 | 15.5 | 8 | 5 | 28.5 |
| 1319 | RR | 10 | 2001 | 25.8 | 4.4 | 9.9 | 14.3 | 3 | 3 | 9.0 |
| 1324 | V | 10 | 2001 | 19.8 | 7.0 | 7.7 | 14.7 | 2 | 2 | 21.0 |
| 531 | E | 10 | 2001 | 14.6 | 6.3 | 10.8 | 17.1 | 0 | 0 |  |
| 44 | UU | 11 | 2001 | 19.5 | 1.8 | 8.0 | 9.8 | 1 | 1 | 6.0 |
| 37 | F | 11 | 2001 | 25.3 | 7.7 | 12.1 | 19.9 | 9 | 7 | 29.7 |
| 1473 | HH | 11 | 2001 | 1.0 | 1.2 | 9.4 | 10.6 | 0 | 0 |  |
| 395 | D | 11 | 2001 | 18.8 | 6.3 | 7.4 | 13.7 | 7 | 7 | 44.9 |
| 1474 | W | 11 | 2001 | 12.9 | 4.0 | 8.9 | 13.0 | 3 | 2 | 15.3 |
| 259 | Y | 11 | 2001 | 17.6 | 5.2 | 7.1 | 12.3 | 4 | 3 | 29.8 |
| 1324 | V | 9 | 2002 | 17.3 | 10.3 | 12.0 | 22.3 | 7 | 7 | 12.7 |
| 531 | E | 9 | 2002 | 18.7 | 4.3 | 11.4 | 15.8 | 1 | 1 | 17.0 |
| 916 | L | 9 | 2002 | 24.7 | 2.4 | 6.2 | 8.7 | 0 | 0 |  |
| 536 | ZZ | 9 | 2002 | 22.1 | 0.9 | 2.1 | 3.0 | 1 | 1 | 37.0 |
| 259 | Y | 9 | 2002 | 18.4 | 7.2 | 5.6 | 12.7 | 7 | 4 | 47.3 |
| 37 | F | 9 | 2002 | 25.5 | 8.4 | 13.8 | 22.2 | 26 | 9 | 16.0 |
| 395 | D | 10 | 2002 | 23.0 | 5.5 | 7.0 | 12.6 | 3 | 2 | 40.7 |
| 1480 | W | 11 | 2002 | 32.6 | 0.2 | 7.9 | 8.1 | 0 | 0 |  |
| 1469 | W | 11 | 2002 | 31.8 | 0.1 | 5.6 | 5.7 | 0 | 0 |  |
| 1319 | RR | 11 | 2002 | 33.9 | 12.1 | 12.1 | 24.2 | 9 | 9 | 23.3 |
| 924 | XX | 11 | 2002 | 7.0 | 2.6 | 2.6 | 5.1 | 0 | 0 |  |
| 1324 | V | 11 | 2002 | 31.4 | 5.4 | 15.0 | 20.4 | 5 | 5 | 33.6 |
| 531 | E | 11 | 2002 | 39.4 | 4.4 | 11.5 | 15.9 | 6 | 6 | 23.8 |
| 916 | L | 11 | 2002 | 43.4 | 0.4 | 5.2 | 5.6 | 0 | 0 |  |
| 259 | Y | 11 | 2002 | 42.3 | 3.6 | 5.3 | 8.9 | 4 | 4 | 36.0 |
| 536 | ZZ | 11 | 2002 | 40.9 | 0.4 | 2.7 | 3.1 | 0 | 0 |  |
| 921 | MM | 11 | 2002 | 33.8 | 1.5 | 4.3 | 5.8 | 2 | 2 | 39.5 |
| 536 | ZZ | 10 | 2003 | 25.8 | 2.8 | 6.1 | 8.8 | 3 | 2 | 12.3 |
| 921 | MM | 10 | 2003 | 34.6 | 1.0 | 4.1 | 5.1 | 3 | 1 | 20.0 |
| 1469 | W | 11 | 2003 | 22.2 | 1.7 | 4.3 | 6.0 | 0 | 0 |  |
| 536 | ZZ | 9 | 2004 | 22.1 | 5.4 | 4.5 | 9.9 | 5 | 4 | 23.4 |
| 916 | L | 9 | 2004 | 33.4 | 1.9 | 8.5 | 10.4 | 4 | 2 | 10.5 |
| 1480 | W | 9 | 2004 | 11.9 | 2.2 | 9.2 | 11.4 | 1 | 1 | 10.0 |
| 1324 | V | 9 | 2004 | 18.4 | 5.5 | 11.5 | 17.0 | 6 | 5 | 7.2 |
| 921 | MM | 11 | 2004 | 37.0 | 1.2 | 3.3 | 4.5 | 3 | 1 | 14.0 |
| 1469 | W | 11 | 2004 | 26.5 | 1.2 | 3.1 | 4.3 | 0 | 0 |  |
| 1178 | RR | 11 | 2004 | 27.3 | 0.6 | 7.2 | 7.9 | 1 | 1 | 50.0 |
| 1328 | B | 11 | 2004 | 22.4 | 1.5 | 4.9 | 6.4 | 2 | 2 | 54.0 |
| 71 | Y | 11 | 2004 | 22.1 | 1.0 | 5.5 | 6.6 | 1 | 1 | 21.0 |
| 536 | ZZ | 11 | 2004 | 47.4 | 1.9 | 2.9 | 4.8 | 2 | 2 | 34.0 |
| 531 | E | 11 | 2004 | 21.6 | 7.7 | 3.2 | 10.8 | 12 | 8 | 37.3 |
| 1324 | V | 9 | 2005 | 11.4 | 6.7 | 7.9 | 14.6 | 8 | 5 | 38.1 |
| 814 | CD | 9 | 2005 | 9.6 | 4.7 | 5.3 | 10.0 | 3 | 1 | 15.3 |
| 916 | L | 9 | 2005 | 23.2 | 7.1 | 6.4 | 13.5 | 8 | 5 | 21.1 |
| 1469 | W | 11 | 2005 | 6.4 | 1.3 | 5.6 | 6.9 | 3 | 1 | 50.7 |
| 1480 | W | 11 | 2005 | 37.0 | 10.3 | 16.1 | 26.4 | 22 | 10 | 16.8 |
| 531 | E | 11 | 2005 | 13.3 | 8.4 | 8.7 | 17.1 | 6 | 3 | 37.5 |
| 1324 | V | 11 | 2005 | 28.1 | 6.7 | 8.4 | 15.1 | 19 | 6 | 21.8 |
| 395 | D | 11 | 2005 | 19.3 | 6.1 | 3.3 | 9.4 | 5 | 2 | 8.0 |
| 814 | CD | 11 | 2005 | 32.4 | 4.4 | 5.0 | 9.4 | 5 | 4 | 17.0 |
| 1480 | W | 9 | 2006 | 20.1 | 15.1 | 14.2 | 29.3 | 6 | 5 | 27.5 |
| 433 | D | 9 | 2006 | 7.6 | 1.5 | 4.6 | 6.2 | 2 | 1 | 39.0 |
| 711 | F | 9 | 2006 | 9.5 | 0.3 | 2.5 | 2.8 | 0 | 0 |  |
| 1324 | V | 9 | 2006 | 18.1 | 2.6 | 14.7 | 17.2 | 2 | 2 | 11.0 |
| 290 | B | 9 | 2006 | 11.2 | 2.5 | 5.0 | 7.5 | 5 | 2 | 43.0 |
| 1178 | RR | 9 | 2006 | 13.1 | 3.5 | 14.0 | 17.4 | 2 | 2 | 39.0 |
| 916 | L | 9 | 2006 | 13.1 | 8.9 | 4.6 | 13.5 | 5 | 3 | 47.0 |
| 921 | MM | 11 | 2006 | 33.5 | 9.5 | 11.7 | 21.2 | 2 | 1 | 7.0 |
| 1469 | W | 11 | 2006 | 27.5 | 6.8 | 7.2 | 14.0 | 5 | 5 | 7.0 |
| 71 | Y | 11 | 2006 | 16.7 | 1.7 | 6.4 | 8.1 | 1 | 1 | 74.0 |
| 1480 | W | 11 | 2006 | 21.3 | 12.7 | 17.2 | 29.9 | 2 | 2 | 52.0 |
| 1494 | ST | 11 | 2006 | 29.0 | 4.6 | 3.4 | 7.9 | 0 | 0 |  |
| 1324 | V | 11 | 2006 | 21.8 | 3.5 | 10.1 | 13.6 | 3 | 2 | 52.7 |
| 1495 |  | 11 | 2007 | 25.1 | 10.0 | 15.6 | 25.6 | 19 | 7 | 31.9 |
| 814 | CD | 11 | 2007 | 29.3 | 10.0 | 9.4 | 19.4 | 17 | 9 | 40.2 |
| 433 | D | 9 | 2008 | 1.8 | 1.9 | 6.7 | 8.6 | 0 | 0 |  |
| 1501 | BB | 9 | 2008 | 12.3 | 2.1 | 4.2 | 6.3 | 4 | 2 | 61.0 |
| 1511 | QE | 10 | 2008 | 21.4 | 0.6 | 2.5 | 3.1 | 0 | 0 |  |
| 814 | CD | 10 | 2008 | 23.8 | 3.1 | 6.2 | 9.2 | 4 | 2 | 21.3 |
| 1189 | RR | 10 | 2008 | 6.0 | 1.9 | 3.4 | 5.3 | 3 | 2 | 7.7 |
| 1508 | VH | 11 | 2008 | 28.3 | 1.0 | 3.2 | 4.3 | 2 | 2 | 45.5 |
| 711 | F | 11 | 2008 | 39.7 | 4.3 | 3.9 | 8.3 | 1 | 1 | 8.0 |
| 1497 | AZ | 11 | 2008 | 26.2 | 3.4 | 4.1 | 7.6 | 2 | 2 | 7.0 |
| 921 | MM | 11 | 2008 | 39.5 | 4.3 | 4.4 | 8.7 | 7 | 4 | 24.7 |
| 948 | L | 11 | 2008 | 39.0 | 2.0 | 6.9 | 8.9 | 1 | 1 | 0.0 |
| 566 | E | 11 | 2008 | 26.0 | 2.7 | 8.5 | 11.2 | 2 | 2 | 19.0 |
| 216 | ZZ | 11 | 2008 | 24.3 | 1.1 | 3.1 | 4.3 | 0 | 0 |  |
| 1516 | TY | 11 | 2008 | 21.6 | 1.2 | 3.9 | 5.1 | 0 | 0 |  |
| 1501 | BB | 11 | 2008 | 22.2 | 1.3 | 4.0 | 5.3 | 2 | 2 | 26.5 |
| 1511 | QE | 9 | 2009 | 14.0 | 1.6 | 5.6 | 7.1 | 1 | 1 | 31.0 |
| 948 | L | 9 | 2009 | 24.1 | 5.4 | 6.4 | 11.8 | 0 | 0 |  |
| 1508 | VH | 9 | 2009 | 25.6 | 6.5 | 6.1 | 12.6 | 10 | 4 | 44.5 |
| 1516 | TY | 9 | 2009 | 20.1 | 2.2 | 4.7 | 7.0 | 0 | 0 |  |
| 1501 | BB | 9 | 2009 | 19.1 | 10.9 | 12.3 | 23.1 | 3 | 3 | 21.0 |
| 1497 | AZ | 9 | 2009 | 16.1 | 5.0 | 9.4 | 14.3 | 12 | 3 | 48.1 |
| 1511 | QE | 11 | 2009 | 29.1 | 2.7 | 5.4 | 8.1 | 3 | 3 | 26.3 |
| 445 | D | 11 | 2009 | 17.4 | 6.4 | 7.6 | 14.0 | 8 | 5 | 37.0 |
| 1516 | TY | 9 | 2010 | 13.7 | 4.3 | 6.9 | 11.1 | 2 | 1 | 32.0 |
| 1511 | QE | 10 | 2010 | 26.3 | 5.7 | 10.2 | 15.9 | 3 | 2 | 13.7 |
| 948 | L | 11 | 2010 | 22.4 | 12.1 | 15.8 | 27.9 | 16 | 10 | 18.1 |
| 1497 | AZ | 11 | 2010 | 23.3 | 4.4 | 11.6 | 16.0 | 13 | 5 | 31.6 |
| 1681 | F | 9 | 2011 | 11.4 | 6.3 | 7.2 | 13.5 | 1 | 1 | 38.0 |
| 1497 | AZ | 9 | 2011 | 13.8 | 3.4 | 19.1 | 22.5 | 3 | 1 | 47.7 |
| 445 | D | 9 | 2011 | 11.9 | 3.4 | 5.6 | 9.0 | 2 | 1 | 54.0 |
| 1497 | AZ | 11 | 2011 | 20.0 | 4.3 | 17.9 | 22.2 | 7 | 2 | 20.4 |
| 1783 | W | 9 | 2012 | 15.9 | 3.0 | 20.4 | 23.4 | 3 | 3 | 43.0 |
| 2076 | SQ | 10 | 2012 | 9.0 | 2.5 | 11.6 | 14.1 | 0 | 0 |  |
| 445 | D | 10 | 2012 | 11.0 | 8.2 | 10.4 | 18.6 | 2 | 2 | 4.0 |
| 2438 | BB | 9 | 2013 | 11.0 | 7.2 | 14.6 | 21.8 | 0 | 0 |  |
| 2357 | MY | 9 | 2013 | 16.4 | 6.8 | 10.1 | 16.9 | 3 | 3 | 2.0 |
| 1872 | PA | 10 | 2013 | 18.2 | 3.3 | 3.3 | 6.6 | 2 | 2 | 52.0 |
| 2150 | HV | 10 | 2013 | 25.7 | 4.0 | 12.3 | 16.3 | 3 | 2 | 34.3 |
| 2382 | RR | 10 | 2013 | 19.5 | 10.1 | 4.2 | 14.3 | 2 | 1 | 28.5 |
| 445 | D | 11 | 2013 | 19.1 | 7.0 | 4.2 | 11.2 | 1 | 1 | 11.0 |
| 2360 | VH | 9 | 2014 | 20.5 | 0.8 | 2.5 | 3.4 | 0 | 0 |  |
| 1846 | NM | 9 | 2014 | 30.3 | 3.3 | 2.5 | 5.8 | 0 | 0 |  |
| 2560 | QL | 10 | 2014 | 19.5 | 1.0 | 2.1 | 3.1 | 1 | 1 | 9.0 |
| 2642 | L | 10 | 2014 | 11.6 | 2.0 | 5.7 | 7.7 | 0 | 0 |  |
| 2382 | RR | 11 | 2014 | 25.7 | 2.6 | 3.4 | 6.0 | 0 | 0 |  |
| 2805 | UK | 11 | 2014 | 22.4 | 2.8 | 4.6 | 7.4 | 0 | 0 |  |
| 2819 | JX | 11 | 2014 | 19.0 | 2.7 | 3.8 | 6.6 | 4 | 2 | 50.8 |
| 2819 | JX | 9 | 2015 | 15.5 | 4.6 | 10.8 | 15.4 | 4 | 3 | 33.0 |
| 2360 | VH | 9 | 2015 | 19.3 | 7.6 | 11.9 | 19.5 | 4 | 3 | 44.3 |
| 1846 | NM | 9 | 2015 | 8.0 | 1.9 | 6.1 | 8.1 | 0 | 0 |  |
| 2382 | RR | 9 | 2015 | 1.9 | 3.0 | 5.3 | 8.4 | 1 | 1 | 9.0 |
| 2560 | QL | 9 | 2015 | 16.5 | 7.4 | 7.8 | 15.2 | 1 | 1 | 53.0 |
| 2795 | PA | 10 | 2015 | 17.4 | 3.9 | 5.9 | 9.9 | 3 | 3 | 16.0 |
| 2867 | AX | 10 | 2015 | 18.9 | 3.1 | 4.7 | 7.8 | 0 | 0 |  |
| 2879 | SA | 10 | 2015 | 2.2 | 2.1 | 1.7 | 3.8 | 1 | 1 | 41.0 |
| 2438 | BB | 11 | 2015 | 21.0 | 1.6 | 6.1 | 7.7 | 0 | 0 |  |
| 2779 | ZU | 11 | 2015 | 9.0 | 5.9 | 12.8 | 18.7 | 1 | 1 | 56.0 |
| 2727 | W | 11 | 2015 | 16.5 | 0.4 | 4.8 | 5.3 | 0 | 0 |  |

Table S2. Dataset used for the experimental analyses using LMMs to compare the probability of eviction and body condition between fed and control pregnant dominant females.

| Dominant female ID | Treatment | Group | Month | Year | Rainfall (mm) | Weight gain | Number of Subordinate females | Number of Subordinate males | Number of helpers | Number of evictions | Number of females evicted | Day until birth |
| --- | --- | --- | --- | --- | --- | --- | --- | --- | --- | --- | --- | --- |
| 355 | Control | SE | 11 | 2010 | 0.8 | -0.09 | 5.4 | 4.2 | 9.6 | 0 | 0 |  |
| 355 | Fed | SE | 10 | 2011 | 3.6 | -0.01 | 5.0 | 9.7 | 14.7 | 6 | 5 | 8.5 |
| 445 | Control | D | 9 | 2011 | 3.0 | 0.16 | 3.4 | 5.6 | 9.0 | 2 | 1 | 54.0 |
| 445 | Control | D | 10 | 2012 | 11.8 | 0.25 | 8.2 | 10.4 | 18.6 | 2 | 2 | 4.0 |
| 815 | Fed | KU | 9 | 2012 | 1.8 | 0.30 | 9.0 | 14.4 | 23.4 | 18 | 11 | 15.5 |
| 943 | Control | JX | 8 | 2011 | 12.4 | 0.19 | 2.9 | 5.4 | 8.3 | 8 | 5 | 38.3 |
| 943 | Fed | JX | 10 | 2011 | 3.6 | 0.20 | 3.9 | 5.8 | 9.7 | 4 | 3 | 22.5 |
| 1497 | Control | AZ | 9 | 2011 | 3.0 | -0.02 | 3.4 | 19.1 | 22.5 | 3 | 1 | 47.7 |
| 1497 | Control | AZ | 11 | 2011 | 3.6 | 0.05 | 2.6 | 12.8 | 15.4 | 7 | 2 | 20.4 |
| 1502 | Control | BB | 11 | 2010 | 0.8 | 0.22 | 7.9 | 17.4 | 25.3 | 7 | 6 | 27.7 |
| 1502 | Control | BB | 9 | 2011 | 3.0 | 0.15 | 2.9 | 10.4 | 13.3 | 4 | 3 | 5.0 |
| 1502 | Fed | BB | 11 | 2011 | 1.0 | 0.08 | 3.4 | 10.9 | 14.3 | 16 | 5 | 15.6 |
| 1510 | Fed | VH | 11 | 2011 | 3.6 | 0.18 | 6.6 | 9.5 | 16.0 | 11 | 7 | 13.5 |
| 1510 | Control | VH | 11 | 2012 | 14.0 | 0.36 | 8.5 | 20.8 | 29.4 | 8 | 7 | 41.3 |
| 1681 | Control | F | 9 | 2011 | 3.0 | 0.19 | 6.3 | 7.2 | 13.5 | 1 | 1 | 38.0 |
| 1735 | Control | EK | 9 | 2011 | 3.0 | 0.03 | 0.5 | 4.8 | 5.3 | 0 | 0 |  |
| 1735 | Fed | EK | 9 | 2012 | 1.8 | 0.27 | 2.0 | 3.9 | 5.9 | 0 | 0 |  |
| 1735 | Control | EK | 11 | 2013 | 0.0 | 0.39 | 4.8 | 6.7 | 11.5 | 3 | 1 | 35.3 |
| 1783 | Control | W | 9 | 2012 | 2.6 | 0.19 | 3.0 | 20.4 | 23.4 | 3 | 3 | 43.0 |
| 1872 | Control | PA | 9 | 2011 | 3.0 |  | 3.5 | 4.1 | 7.6 | 10 | 4 | 44.1 |
| 1873 | Fed | PA | 9 | 2012 | 0.8 | 0.20 | 2.0 | 3.4 | 5.4 | 0 | 0 |  |
| 2052 | Control | RR | 9 | 2011 | 3.0 | 0.24 | 1.7 | 7.2 | 9.0 | 1 | 1 | 18.0 |
| 2052 | Control | RR | 11 | 2011 | 1.0 | -0.15 | 4.4 | 5.8 | 10.2 | 1 | 1 | 35.0 |
| 2052 | Fed | RR | 9 | 2012 | 1.8 | 0.38 | 4.2 | 9.5 | 13.6 | 7 | 7 | 20.4 |
| 2076 | Control | SE | 10 | 2012 | 1.8 | 0.15 | 2.5 | 11.6 | 14.1 | 0 | 0 |  |
| 2082 | Control | UK | 11 | 2012 | 14.0 | -0.06 | 3.7 | 4.6 | 8.3 | 0 | 0 |  |
| 2082 | Fed | UK | 9 | 2012 | 0.8 | 0.35 | 1.7 | 3.3 | 5.1 | 1 | 1 | 34.0 |
| 2219 | Fed | NE | 11 | 2011 | 1.0 | 0.18 | 4.3 | 3.0 | 7.3 | 4 | 2 | 28.3 |
